# The Role of Fibular Fixation in Distal Tibia-Fibula Fractures: A Meta-Analysis

**DOI:** 10.1155/2021/6668467

**Published:** 2021-02-25

**Authors:** Chengxin Li, Zhizhuo Li, Qiwei Wang, Lijun Shi, Fuqiang Gao, Wei Sun

**Affiliations:** ^1^Department of Orthopedics, Peking University China-Japan Friendship School of Clinical Medicine, 2 Yinghuadong Road, Chaoyang District, Beijing 100029, China; ^2^Department of Orthopedics, Graduate School of Peking Union Medical College, China-Japan Friendship Institute of Clinical Medicine, 2 Yinghuadong Road, Chaoyang District, Beijing 100029, China; ^3^Beijing Key Laboratory of Immune Inflammatory Disease, China-Japan Friendship Hospital, 2 Yinghuadong Road, Chaoyang District, Beijing 100029, China

## Abstract

**Objectives:**

The necessity of fibular fixation in distal tibia-fibula fractures remains controversial. This study aimed to assess its impact on radiographic outcomes as well as rates of nonunion and infection.

**Methods:**

A systematic search of the electronic databases of PubMed, Embase, and Cochrane library was performed to identify studies comparing the outcomes of reduction and internal fixation of the tibia with or without fibular fixation. Radiographic outcomes included malalignment and malrotation of the tibial shaft. Data regarding varus/valgus angulation, anterior/posterior angulation, internal/external rotation deformity, and the rates of nonunion and infection were extracted and then polled. A meta-analysis was performed using the random-effects model for heterogeneity.

**Results:**

Additional fibular fixation was statistically associated with a decreased rate of rotation deformity (OR = 0.13; 95% CI 0.02–0.82, *p*=0.03). However, there was no difference in the rate of malreduction between the trial group and the control group (OR = 0.86; 95% CI 0.27–2.74, *p*=0.80). There was also no difference in radiographic outcomes of varus-valgus deformity rate (OR = 0.17; 95% CI 0.03–1.00, *p*=0.05) or anterior-posterior deformity rate (OR = 0.76; 95% CI 0.02–36.91, *p*=0.89) between the two groups. Meanwhile, statistical analysis showed no significant difference in the nonunion rate (OR = 0.62; 95% CI 0.37–1.02, *p*=0.06) or the infection rate (OR = 0.81; 95% CI 0.18–3.67, *p*=0.78) between the two groups.

**Conclusions:**

Additional fibular fixation does not appear to reduce the rate of varus-valgus deformity, anterior-posterior deformity, or malreduction. Meanwhile, it does not appear to impair the union process or increase the odds of infection. However, additional fibular fixation was associated with decreased odds of rotation deformity compared to controls.

## 1. Introduction

Combined distal tibia and fibula fractures are one of the most common diaphyseal fractures among all long bones. These injuries are caused mainly by high-energy trauma such as motor vehicle accidents or low-energy torsional trauma. With the widespread use of high-speed transport, the incidence of this injury is still increasing [1]. Today, the use of intramedullary (IM) nailing to treat the tibial fracture has been well defined because of the development of newer intramedullary implants and the associated reduction in complications [2]. However, the role of fibular fixation in addition to tibial IM nailing in distal extraarticular tibia-fibular fractures remains controversial [3, 4]. Several studies exploring the effects of fibular fixation on distal tibial fractures have been carried out [5–7]. Studies supporting fibular fixation found that it is related to a better anatomical alignment and better control over rotation while also introducing stability and restoring limb length [8–10]. Additionally, it has been reported that there are significantly higher rates of loss of reduction in distal tibia fractures treated with an IM nail without plate stabilization of the combined fibula fracture [11]. Moreover, after fibula fixation, the biomechanical structure is considered to be more similar to that present before the injury, which will reduce by 1/6 the total load applied to the knee joint [12] and between 6% and 7% of the total load transmitted through both the tibia and fibula [13, 14]. Conversely, the opposing view is that fibular fixation may result in delayed union or nonunion because it inhibits cyclic loading on the tibial fracture site [15, 16]. Meanwhile, high-energy fractures of the distal tibia are often accompanied with a high incidence of soft tissue trauma leading to a high incidence of wound infections and necrosis [17]. Consequently, the open reduction and internal fixation of the fibula required often increases the rate of wound complications [18]. Present, there is no clear consensus on the optimum management of combined distal third tibia and fibula fractures. Our aim was to assess whether combined distal third tibia and fibula fractures will benefit from concurrent fibular fixation.

## 2. Materials and Methods

### 2.1. Search Strategy

A systematic search of PubMed, Embase, and Cochrane library was conducted to identify studies comparing the outcomes with or without fibular fixation in addition to tibial reduction and internal fixation in distal extraarticular tibia-fibular fractures. The following keywords or corresponding Medical Subject Headings (MeSH) were used: “distal tibia and fibular fracture,” “extra-articular fracture,” “fibula fixation,” and “tibia fixation.” The systematic search of medical reference libraries occurred between August 1 through August 15, 2020. Reference lists of related publications (especially reviews and meta-analyses) mentioning the role of fibular fixation were also carefully screened to identify studies that were not captured in our initial database search. There were no language or data restrictions.

### 2.2. Involvement Inclusion and Exclusion Criteria

An article was considered eligible when it concerned (1) distal extraarticular tibia and fibula fractures, (2) fibular fixation versus lack of fibula fixation, (3) closed or open fractures, and (4) radiographic outcomes, nonunion, and infection rate as well as other clinical variables provided as endpoints. Exclusion criteria of this investigation were (1) intraarticular fractures; (2) cadaver studies, animal studies, and other biomechanical studies; (3) case reports, study protocols, letters, correspondence, conference presentations, and noncomparative studies; and (4) studies that did not report on the primary outcomes, radiographic, and/or functional outcomes. We first removed redundant and unrelated records by reading the titles and abstracts. Then, full texts of the remaining articles were downloaded to confirm their eligibility based on the above criteria.

### 2.3. Data Extraction

Two reviewers reviewed and extracted data from studies that fulfilled all inclusion and exclusion criteria. The following variables were extracted from each study: author's name, year of study, type of study, level of evidence, demographic data, and type of surgery.

### 2.4. Patient and Public Involvement

No patients were involved in this study.

### 2.5. Assessment of Methodological Quality and Risk of Bias of the Study

The assessments of each of the studies selected for the final analysis were performed independently by two reviewers. A 12-item scale [19] was used to assess the methodological quality of each included study. The 12-item scale consisted of the following: adequate randomization, concealment of allocation, patient blinding, care provider blinding, outcome assessor blinding, dropout rate, intention-to-treat (ITT) analysis, avoidance of selective reporting, similarity of baseline characteristics, similarity or absence of cofactors, patient compliance, and similarity of timing. Any disagreement was resolved by discussion and consensus.

### 2.6. Statistical Analysis

Statistical analyses were performed using the random-effects model with inverse variance weighting. Meta-analyses and forest plots were constructed with the statistical software Review Manager (RevMan) ((computer program) Version 5.3. Copenhagen: The Nordic Cochrane Centre, The Cochrane Collaboration, 2014). For binary data, pooled odds ratios (OR) as well as related 95% confidence intervals (CIs) were adopted, and a pooled 95% CI not covering 1 indicated a significant difference between the two groups; meanwhile, pooled weighted mean difference (WMD) or standardized mean difference (SMD), as well as related 95% CIs, were used to evaluate continuous data, and a 95% CI not covering 0 revealed a significant difference. Heterogeneity was evaluated between individual studies with a Q statistic and *I*^2^ value for each meta-analysis. *I*^2^ heterogeneity less than 25% generally indicates consistent results and homogenous studies, while 25–75% indicates moderate heterogeneity and greater than 75% indicates severe heterogeneity. If *I*^2^ was >50%, sensitivity analyses were conducted by omitting one study at a time to examine the influence of each.

## 3. Results

### 3.1. Description of Included Studies

**Figure 1:**
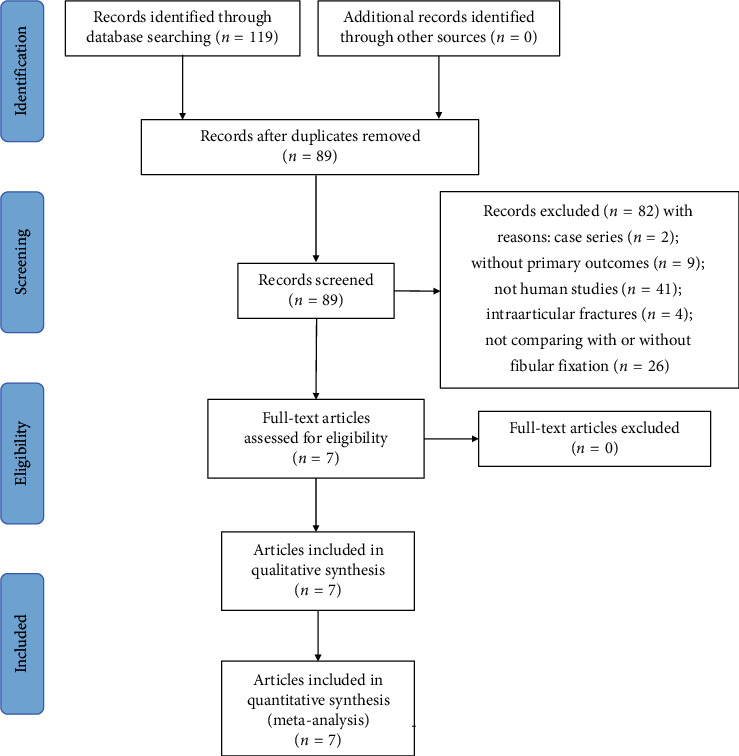
Flow diagram of literature research and the selection process.

Overall, the initial search yielded 119 potentially relevant articles: 86 from PubMed and 33 from Embase. Of these, 30 duplicates were removed using Endnote software. After reading the titles and abstracts of the 89 remaining articles, 82 were excluded. Therefore, seven studies [20–26] fulfilled all inclusion and exclusion criteria and were included in this systematic review and meta-analysis. The inclusion processes and reasons for exclusion are depicted in Figure 1. Three [22, 24, 25] studies were randomized controlled trials, another three [20, 21, 26] of the seven were retrospective studies, and only one [23] was a prospective cohort study. All patients, both in the trial and control groups, were treated with interlocking IM nail or plate fixation for tibia fracture, and patients in the trial group additionally underwent fixation with a 3.5 mm dynamic compression plate (DCP) for fibula fracture. Two studies [22, 23] excluded patients with open fractures, leaving all patients included with closed fractures. All patients were followed up for more than 6 months (from 6 to 21 months) with valgus/varus and posterior/anterior angulations and nonunion, and the infection rates of most patients were assessed. Two studies [23, 24] mentioned the functional outcomes or the range of movements at the ankle. Two studies [20, 21] only reported the nonunion rate or union of time. The main characteristics of the selected studies are listed in Table 1.

As for the risk of bias (RoB) of the included articles, only if the method of randomizing was explicitly described and the dropout rate was <20%, was the study given a score of “1;” otherwise, the score was “0.” For ITT, only if all randomized participants were analyzed in the group, were they allocated to the study and received a “1” score. If the studies met at least 6 of the 12 criteria, the study was regarded as having low RoB. If five or fewer of the 12 criteria were met, the study was labeled as high RoB. Results of the RoB assessment are summarized in Table 2.

### 3.2. Quantitative Analysis

#### 3.2.1. Nonunion Rate at 6 Months after Surgery

Radiographic union was defined as cortical bridging on three or more cortices on orthogonal radiographic views. Nonunion was defined as a fracture with no radiographic progression toward healing at 9 months after surgery on consecutive radiographs over a minimum 2-month period accompanied by clinical symptoms of nonunion (pain, inability to bear weight). Delayed union was defined using the same definition, but for fractures between 6 and 9 months.

**Figure 2:**
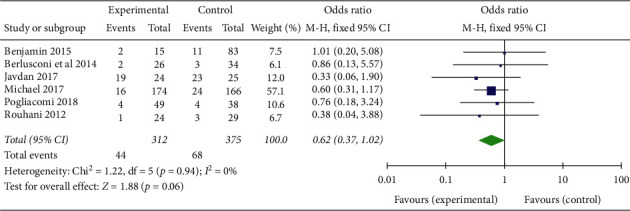
Nonunion rate at 6 months after surgery.

Based on six comparative studies, the statistical results (OR = 0.62; 95% CI 0.37–1.02, *p*=0.06; *I*^2^ = 0%, *p* for heterogeneity = 0.94) suggested no differences in the nonunion rate at 6 months after surgery between the trial group and the controls (Figure 2).

#### 3.2.2. Varus-Valgus Deformity

**Figure 3:**
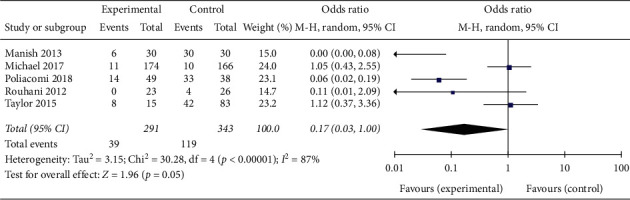
Varus-valgus deformity.

Varus-valgus deformity was measured on the anteroposterior projections by determining the angle formed by the intersection between the perpendicular lines drawn from the tibial plateau and the tibia plafond. Varus-valgus deformity was defined as coronal plane deviation >5° on final radiographs. From five comparative studies, there was no difference in varus-valgus deformity rates between patients in the trial group and controls (OR = 0.17; 95% CI 0.03–1.00, *p*=0.05; *I*^2^ = 87%, *p* < 0.00001 for heterogeneity) (Figure 3).

#### 3.2.3. Anterior-Posterior Deformity

**Figure 4:**
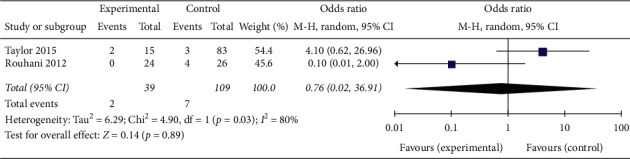
Anterior-posterior deformity.

Anterior-posterior deformity was defined as sagittal plane deviation >10° on the final radiograph. From two comparative studies, the pooled results (OR = 0.76; 95% CI 0.02–36.91, *p*=0.89; *I*^2^ = 80%, *p*=0.03 for heterogeneity) showed no significant difference in anterior-posterior deformity rates between patients in the trial group and controls (Figure 4).

#### 3.2.4. Rotational Deformity

By standing at the foot end of the patient, the rotation of the ankle was determined by measuring the angle subtended by a plumb line with a line passing through the midpoint of the knee, the line joining the midpoint of the ankle (intermalleolar distance) and the second toe. Rotation deformity was defined as an internal/external rotation deformity >10° compared to the normal contralateral limb.

**Figure 5:**
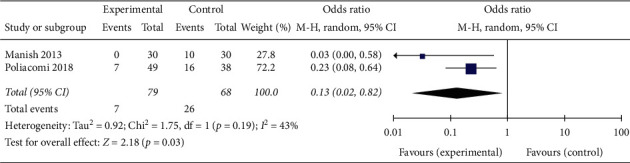
Rotational deformity.

The pooled results (OR = 0.13; 95% CI 0.02–0.82, *p*=0.03; *I*^2^ = 43%, *p*=0.019 for heterogeneity) of two comparative studies suggested that fibular fixation was associated with decreased odds of rotational deformity (Figure 5).

#### 3.2.5. Malreduction

**Figure 6:**
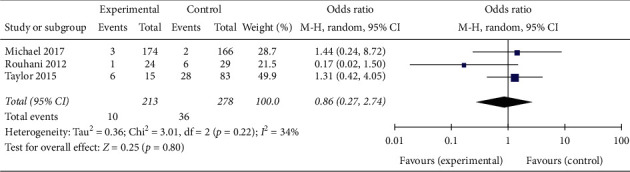
Malreduction.

Malreduction was defined as coronal or sagittal plane deviation of >5° on immediate postoperative radiographs. The pooled results (OR = 0.86; 95% CI 0.27–2.74, *p*=0.80; *I*^2^ = 34%, *p*=0.22 for heterogeneity) of three comparative studies suggested that there was no significant difference in malreduction rates between patients in the trial group and controls (Figure 6).

#### 3.2.6. Rate of Infection

**Figure 7:**
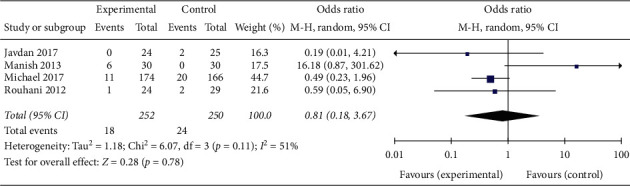
Rate of infection.

Six of the studies mentioned the adverse event of infection, but only five provided data and one of the studies noted that there were no infection cases in either of the groups. The results (OR = 0.81; 95% CI 0.18–3.67, *p*=0.78; *I*^2^ = 51%, *p*=0.11 for heterogeneity) of the other four studies showed that there was no significant difference in infection rates between patients in the trial group and controls (Figure 7).

## 4. Discussion

With the frequent occurrence of traffic accidents, fractures of the distal tibia and fibula are common in this population. Most patients who suffer from this high-energy injury need surgical intervention. However, as a result of the low soft tissue coverage and poor blood supply of the distal tibia, the incidences of delayed union or nonunion and other complications are high. To reduce these complications and improve prognosis, the surgical options to treat distal tibia and fibular fractures have significantly evolved over the past several decades. In 1969, a precedent for fixation of the fibula associated with distal tibia intraarticular fractures was established by Ruedi and Allgower [27]. They advocated that internal fixation was feasible for distal tibiofibular fractures within 10 cm of the ankle joint. However, the need for fibular fixation in distal tibia extraarticular fractures is not clear.

Cadaveric biomechanical experiments designed to investigate the value of adjunctive fibular fixation with tibial fixation have vastly contributed to this subject. Strauss et al. [6] conducted a laboratory experiment to compare IM nails with locked plates in the treatment of tibia fractures with concurrent same level fibula fractures. They found that an imperfect fibula achieved by osteotomy significantly increased the risk of construct displacement, regardless of which type of fixation was used. Therefore, the authors concluded that an intact fibula may improve the fracture fixation stability of the distal tibia. Another cadaveric study designed by Kumar et al. [3] investigated the effect of fibular plate fixation on axial rotation of simulated distal fractures of the tibia and fibula. They created a 5 mm transverse segmental defect which was 7 cm proximal to the ankle joint at the same level in the tibia and fibula and then used a 9 mm Russell-Taylor IM nail to fix the tibia. They also found that additional fibular plate fixation decreased axial rotation and increased the rotational stability, but did not increase rotational stiffness. However, Weber et al. [5] reported that the effect of fibular plate fixation on stability was weakened if the tibia was fixed with an IM nail.

According to our results, there were no significant differences in varus-valgus deformity, anterior-posterior deformity, and malreduction rate, and only the rotation deformity rate was significantly reduced. Usually, studies assessed distal tibia fibular fracture malalignment after IM nailing with and without fibular stabilization at two different times: immediately after surgery and again at regular follow-up after surgery. The initial alignment immediately after surgery represents the result of reduction. There were no significant differences regarding the malreduction in our study, which indicated that fibular fixation does not affect the surgery of the tibia. However, during the follow-up, the rate of rotation deformities was significantly reduced, which suggested that instability was associated with the lack of fibular fixation. Several groups of studies have reported that fibular fixation preserved the reduction of the tibia in the same level in combined tibial and fibular fractures and have suggested concurrent fibular fixation [11]. Kumar et al. reported that fibular plate fixation increased the initial rotational stability after distal tibial fracture in comparison with patients that were treated by tibial IM nailing alone, which correlated with our findings. Others have also mentioned that the highest rate of complications was seen in fibular distal fractures without fibular additional plating and recommended fibular fixation in combined tibial and fibular fractures [13, 14]. Regarding tibiofibular stability, fibular fixation is advisable to avoid rotational deformity. All seven studies included in this study involved a fibular fixation treatment group and a control group. To evaluate union, we adopted the nonunion rate at 6 months to assess the results. All studies reported the outcomes, and the incidence of nonunion ranged from 0 to 79% in the treatment group and from 0 to 92% in the control group. The pooled results (OR = 0.62; 95% CI 0.37–1.02; *p*=0.06) suggested no significant differences in the nonunion rate at 6 months.

Sensitivity analysis showed that there was no change in the results with the removal of any set of data. We also examined the rate of infection, which is another complication that may relate to the surgery. The results (OR = 0.81; 95% CI 0.18–3.67; *p*=0.78) also do not support the theory that supplementary fibular fixation increases the rate of infection. There have been previous reports that additional surgical procedures can destroy surrounding tissues and blood supply, which play important roles in fracture healing [15, 16]. However, the fact of the combined fracture itself suggests relatively high-energy trauma and a high incidence of complications such as delayed union and infection. The results of two studies which excluded patients with open fractures are similar to our meta-analysis. These results do not support the hypothesis that adjunctive fibular fixation can increase the rate of nonunion and infection. Additionally, the malalignment rate is another series of outcomes that we intend to compare.

To our knowledge, this is the first meta-analysis comparing tibia fixation with or without fibular fixation. However, there are also some limitations as follows. First, as only three RCTs in this area have been identified, our study included four non-RCTs, which inevitably involved selection, recall, and interviewer bias, thus eventually weakening our results. Second, patients were enrolled in every study according to different criteria; some excluded all open fractures, while some included both open and closed fractures, creating significant heterogeneity in wound healing and infection rates. Moreover, there is a lack of a uniform method to treat tibia fractures or fibula fractures. None of the studies noted the reason for the choice of IM nailing or plate, which can influence the clinical outcomes. Finally, the follow-up duration was relatively short, preventing examination of long-term outcomes, especially postoperative function.

## 5. Conclusions

According to our systematic review and meta-analysis, we can conclude that additional fibular fixation does not appear to reduce the rates of varus-valgus deformity, anterior-posterior deformity, or malreduction. Moreover, neither does it appear to impair the union process or increase the odds of infection. However, additional fibular fixation was associated with decreased odds of rotation deformity compared to controls.

## Figures and Tables

**Table 1:** The main characteristics of selected studies.

Author	Years	Type of design	Case	Average	Open fracture: closed fracture	Type of surgery	Average follow-up	Assessment
Francesco et al. [23]	2018	Prospective cohort study	Fibular fixation: 49 No fibular fixation: 38	Fibular fixation: 56.4 No fibular fixation: 59.8	No open fractures	Interlocking intramedullary nail for tibia and plating fixation for fibular	18 months	Rotational alignment and valgus/varus and posterior/anterior angulations
Michael et al. [21]	2017	Retrospective study	Fibular fixation: 166 No fibular fixation: 174	Fibular fixation: 166 No fibular fixation: 174	Fibular fixation: 93 : 73 No fibular fixation: 95 : 79	Interlocking intramedullary nail for tibia and plating fixation for fibular	21 months	Time to union, delayed union, and nonunion
Mohammad et al. [22]	2017	Randomized controlled study	Fibular fixation: 24 No fibular fixation: 25	Fibular fixation: 36.9 No fibular fixation: 34.8	No open fractures	Interlocking intramedullary nail for tibia and 3.5 mm DCP fixation for fibula	9 months	Valgus/varus and posterior/anterior angulations and nonunion
Benjamin et al. [26]	2015	Retrospective study	Fibular fixation: 15 No fibular fixation: 83	Fibular fixation: 42.8 No fibular fixation: 40.3	Fibular fixation: 1 : 14 No fibular fixation: 30 : 53	Interlocking intramedullary nail for tibia and plating fixation for fibular	117 months	Valgus/varus and posterior/anterior angulations and nonunion
Berlusconi et al. [20]	2014	Retrospective study	Fibular fixation: 26 No fibular fixation: 34	Fibular fixation: 47.12 No fibular fixation: 44	Fibular fixation: 9 : 17 No fibular fixation: 10 : 24	Interlocking intramedullary nail for tibia and plating fixation for fibular	>6 months	Nonunion
Manish et al. [24]	2013	Randomized controlled study	Fibular fixation: 30 No fibular fixation: 30	NA	Fibular fixation: 14 : 16 No fibular fixation: 12 : 18	Interlocking intramedullary nail for tibia and 3.5 mm DCP fixation for fibula	18 months	Rotational alignment and valgus/varus and posterior/anterior angulations
Rouhani et al. [25]	2012	Randomized controlled study	Fibular fixation: 24 No fibular fixation: 29	Fibular fixation: 24.2 No fibular fixation: 28.6	Fibular fixation: 11 : 13 No fibular fixation: 17 : 12	Interlocking intramedullary nail for tibia and 3.5 mm DCP fixation for fibula	6 months	Valgus/varus and posterior/anterior angulations and nonunion

NA, not available.

**Table 2:** Results of the risk of bias assessment.

Study	Randomized adequately	Allocation concealed	Patient blinded	Care provider blinded	Outcome assessor blinded	Acceptable dropout rate	ITT analysis	Avoided selective reporting	Similar baseline	Similar on avoided cofactor	Patient compliance	Similar timing	Total score	Risk of bias
Francesco et al. [23]	0	0	0	0	0	1	0	1	1	1	1	1	6	Low
Michael et al. [21]	0	0	0	0	0	1	0	1	1	1	1	1	6	Low
Mohammad et al. [22]	1	0	0	0	0	1	0	1	1	1	1	1	7	Low
Benjamin et al. [26]	0	0	0	0	0	1	0	1	1	1	1	1	6	Low
Berlusconi et al. [20]	0	0	0	0	0	1	0	1	1	1	1	1	6	Low
Manish et al. [24]	1	1	0	0	0	1	0	1	1	1	1	1	8	Low
Rouhani et al. [25]	1	1	0	0	0	1	0	1	1	1	1	1	8	Low

## Data Availability

The datasets used and/or analyzed during the present study are available from the corresponding author upon request.
